# Genetic and epigenetic factors of arterial hypertension: a bibliometric- and in-silico-based analyses

**DOI:** 10.3389/fmolb.2023.1221337

**Published:** 2023-10-12

**Authors:** Raushan Zh Karabaeva, Tamara A. Vochshenkova, Afshin Zare, Nazanin Jafari, Hanieh Baneshi, Nadiar Maratovich Mussin, Rustam Kuanyshbekovich Albayev, Asset Askerovich Kaliyev, Akmaral Baspakova, Amin Tamadon

**Affiliations:** ^1^ Gerontology Center, Medical Center of the President’s Affairs Administration of the Republic of Kazakhstan, Astana, Kazakhstan; ^2^ Therapeutic Department, Asfendiyarov Kazakh National Medical University, Almaty, Kazakhstan; ^3^ PerciaVista R&D Co., Shiraz, Iran; ^4^ General Surgery, West Kazakhstan Marat Ospanov Medical University, Aktobe, Kazakhstan; ^5^ Department for Scientific Work, West Kazakhstan Marat Ospanov Medical University, Aktobe, Kazakhstan

**Keywords:** arterial hypertension, epigenetic factors, genetic factors, miRNA, *in silico* analyses

## Abstract

**Introduction:** Arterial hypertension (AH) is a pervasive global health concern with multifaceted origins encompassing both genetic and environmental components. Previous research has firmly established the association between AH and diverse genetic factors. Consequently, scientists have conducted extensive genetic investigations in recent years to unravel the intricate pathophysiology of AH.

**Methods:** In this study, we conducted a comprehensive bibliometric analysis employing VOSviewer software to identify the most noteworthy genetic factors that have been the focal point of numerous investigations within the AH field in recent years. Our analysis revealed genes and microRNAs intricately linked to AH, underscoring their pivotal roles in this condition. Additionally, we performed molecular docking analyses to ascertain microRNAs with the highest binding affinity to these identified genes. Furthermore, we constructed a network to elucidate the in-silico-based functional interactions between the identified microRNAs and genes, shedding light on their potential roles in AH pathogenesis.

**Results:** Notably, this pioneering *in silico* examination of genetic factors associated with AH promises novel insights into our understanding of this complex condition. Our findings prominently highlight miR-7110-5p, miR-7110-3p, miR-663, miR-328-3p, and miR-140-5p as microRNAs exhibiting a remarkable affinity for target genes. These microRNAs hold promise as valuable diagnostic and therapeutic factors, offering new avenues for the diagnosis and treatment of AH in the foreseeable future.

**Conclusion:** In summary, this research underscores the critical importance of genetic factors in AH and, through *in silico* analyses, identifies specific microRNAs with significant potential for further investigation and clinical applications in AH management.

## 1 Introduction

**FIGURE 1:**
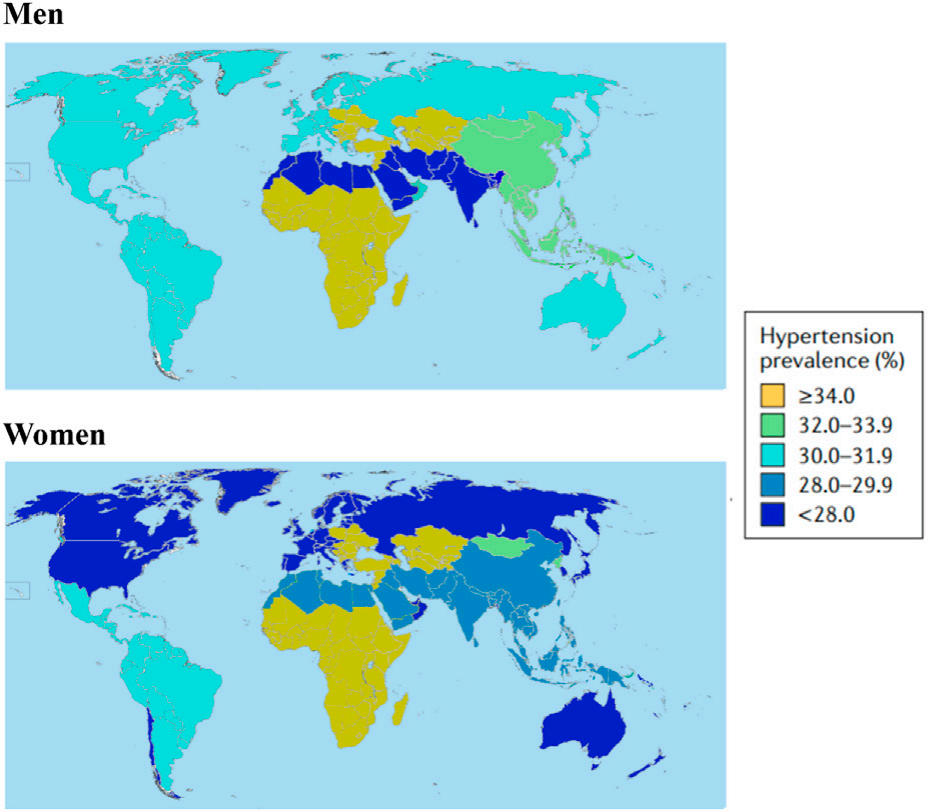
Hypertension prevalence all over the world in 2010. The definition of prevalence of hypertension is systolic blood pressure≥140 mmHg or diastolic blood pressure≥90 mmHg or use of antihypertensive drugs in men and women. Data obtained from Mills et al. (2016).

Arterial hypertension (AH) stands as a significant global public health concern, impacting millions of individuals worldwide (Lauder et al., 2020) (Figure 1). As of the most recent statistics available, which were obtained from Whelton et al. (2023), AH affects a substantial portion of the global population. The prevalence of AH varies across regions, with high-income countries reporting 28.5% (Mills et al., 2016) and low- and middle-income countries experiencing 17.5% (Geldsetzer et al., 2019) of the cases.

The majority of hypertensive patients exhibit modifiable cardiovascular risk factors that contribute to the development and progression of AH (Mills et al., 2016). These risk factors encompass elements such as overweight, diabetes mellitus (DM), smoking habits, a sedentary lifestyle, and high salt intake (Pinto and Martins, 2017). Therefore, uncovering the underlying etiology of AH remains essential. Although the precise etiology of AH remains elusive, previous research has emphasized the intricate interplay between environmental and genetic factors (Dornas and Silva, 2011).

In addition to environmental factors, genetic predisposition plays a pivotal role in the development of AH. Several genetic factors have been identified, some of which predispose individuals to AH, while others reduce the risk of the disease. For instance, mutations in eight genes have been associated with Mendelian forms of hypertension (Raina et al., 2019). Moreover, recent molecular studies have revealed associations between specific gene polymorphisms and AH, such as those in angiotensinogen (AGT), angiotensin-converting enzyme (ACE), angiotensin (AT) II receptor (type 1), and aldosterone synthase genes (Ghafar, 2020).

Furthermore, in recent years, the role of epigenetic modifications in the pathophysiology of AH has garnered increasing attention (Mao et al., 2023). Epigenetic modifications encompass alterations in gene expression regulation that are heritable, including DNA methylation, histone modifications, and microRNA regulation, without any changes in the nucleotide sequence. Notably, DNA methylation, a key epigenetic modification, can impact various aspects of AH, including renal sodium reabsorption, cardiac hypertrophy, activation of the renin-angiotensin-aldosterone system (RAAS), ionic transport, vascular tone modulation, and more, by affecting various genes (Takeda et al., 2021).

MicroRNAs, as another class of genetic factors, have emerged as important regulators in the pathophysiology of AH (Wang et al., 2022). Previous studies have demonstrated that these non-coding RNAs play a pivotal role in AH through mechanisms such as inflammation regulation, vasodilatory activity, endothelial proliferation, regulation of endothelial nitric oxide synthase (eNOS) levels, and involvement in the differentiation of stem cells into vascular smooth muscle cells (VSMCs) (Aryal and Suárez, 2019). Furthermore, microRNAs have the potential to influence both the RAAS and the sympathetic nervous system, bridging the gap between these two systems and exerting their effects on AH in various ways (Improta-Caria et al., 2021).

Despite extensive research on genetic factors in AH, a deeper understanding of their roles is necessary. In this study, we aim to identify and analyze various genetic factors involved in AH using bibliometric analysis and conduct molecular docking analysis to elucidate their potential functions in the disease. Additionally, we will explore the role of genetic variants and polymorphisms in AH. Our *in silico* analysis will help identify microRNAs with the highest affinity for genes implicated in AH. This analysis may contribute to a more comprehensive understanding of the mechanisms underlying AH and may have implications for future diagnostic and therapeutic research.

In summary, our study underscores the significance of comprehending the genetic factors at play in AH, including the role of microRNAs and epigenetic modifications. We aim to shed light on these factors’ contributions to AH pathogenesis and their potential utility in future diagnostic and therapeutic strategies.

## 2 Materials and methods

### 2.1 Data extraction and bibliometric analysis

To conduct a bibliometric analysis of recent genetic studies related to arterial hypertension (AH), we searched the PubMed online database on 25 April 2023. The search strategy used was: “Search: {[arterial hypertens*(Title/Abstract)] OR [arterial hypertens*(MeSH Terms)]} AND {[gene (MeSH Terms)] OR [gene (Title/Abstract)]} Filters: from 2018–2023”. We used VOSviewer software to perform the bibliometric analysis and identify the most frequent genetic-related MeSH keywords (Van Eck and Waltman, 2010). We excluded some general MeSH keywords from their analysis, including human, male, female, hypertension, blood pressure, familial primary pulmonary hypertension, all Mesh keywords that belong to animals, all Mesh keywords that belong to age, cell, cultured, endothelial cells, muscle and smooth muscle, lung, hypoxia and biomarkers. We also performed an Overlay visualization using VOSviewer to determine the time range in which the most frequent genetic-related MeSH keywords were studied.

The use of bibliometric analysis has become increasingly popular in biomedical research for identifying research trends, and hot topics. In previous studies, bibliometric analysis has been used to investigate the global trends and research focus of hypertension, identifying key research areas and influential authors (Donthu et al., 2021). In addition, network visualization analysis has been used to visualize the co-citation of hypertension-related genes and pathways. By using these methods, researchers have been able to identify potential therapeutic targets and biomarkers for hypertension.

### 2.2 MicroRNA and gene related data extraction

We conducted a deep search in Google Scholar on 26 April 2023, to identify microRNAs and genes involved in AH. They used the following search strategies: 1) allintitle: hypertension microRNA “arterial hypertension” -review–overview, and 2) allintitle: hypertension “gene” “arterial hypertension” -review -overview.

### 2.3 Docking analysis of microRNAs and target genes

We obtained the structure of microRNAs and genes from mirBASE (Kozomara and Griffiths-Jones, 2014) and the National Center for Biotechnology Information (NCBI) (Sayers et al., 2021), respectively. They then performed a docking analysis of microRNAs and target genes using RNAhybrid (Rehmsmeier et al., 2004).

Molecular docking is a computational method that has been used to predict the binding affinity of small molecules to target proteins (Fan et al., 2019). In recent years, molecular docking has been applied to predict the binding of microRNAs to their target genes (Zhou et al., 2022). This approach has been used to identify potential miRNA-gene interactions involved in the pathogenesis of hypertension. By using RNAhybrid for docking analysis of microRNAs and target genes, we can identify potential regulatory mechanisms of the genes involved in AH.

### 2.4 Network visualization

Cytoscape software (Shannon et al., 2003) was utilized in order to visualize Network between microRNAs and genes. Network visualization has been used to visualize complex interactions between genes, microRNAs, and other biomolecules. In previous studies, network visualization has been used to identify key pathways and molecular interactions involved in the pathogenesis of hypertension. By using Cytoscape for network visualization, we can identify potential regulatory networks of microRNAs and genes involved in AH, potentially identifying new therapeutic targets and diagnostic biomarkers.

## 3 Results

### 3.1 The most frequent mesh keywords that are examined in studies in which the roles of genetic factors in arterial hypertension (AH) are studied

**FIGURE 2:**
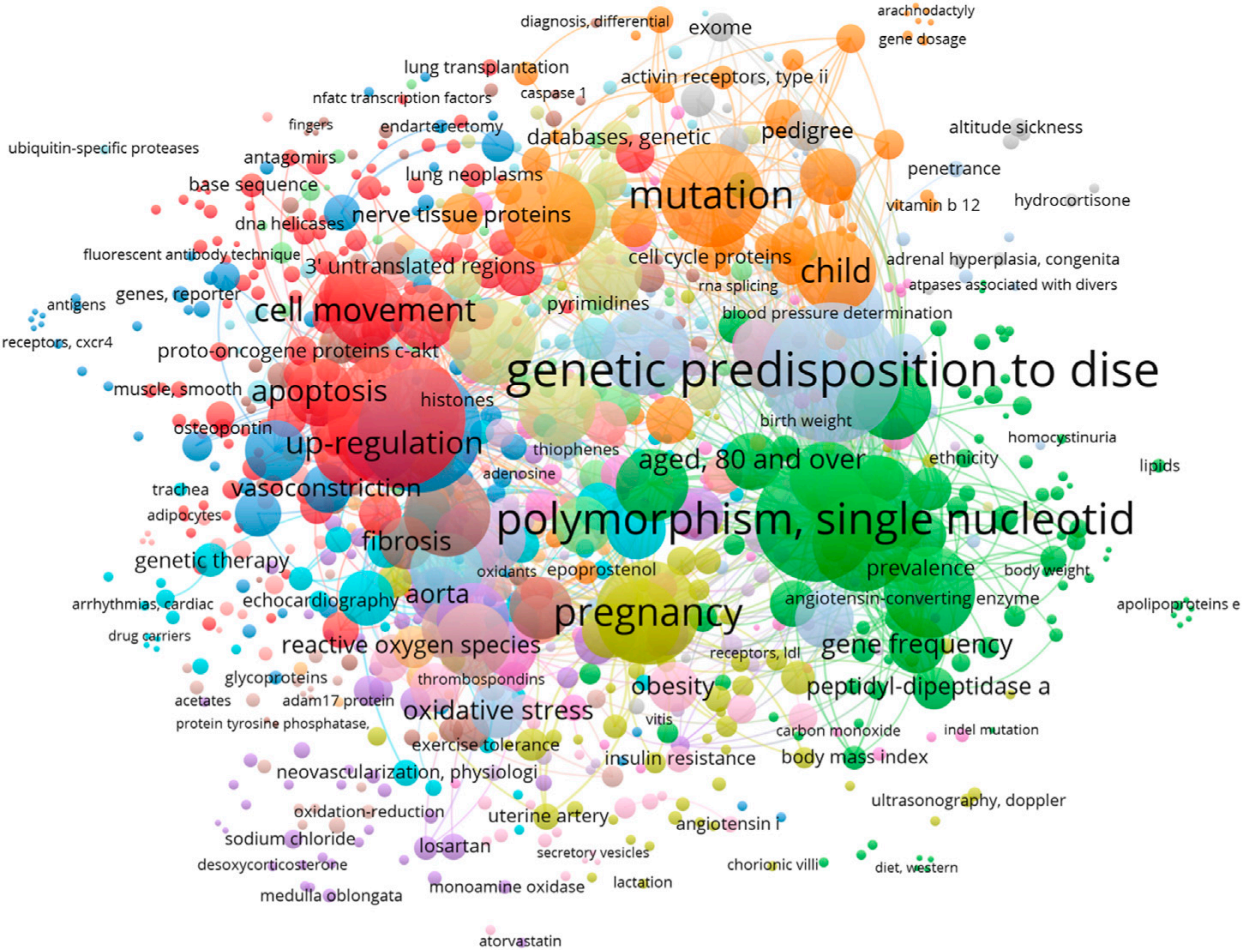
Network visualization of the most frequent Mesh keywords that have examined in studies in which the roles of genetic factors in arterial hypertension (AH) are studied from 2018 to 2023. The size of each square of each MeSH keyword demonstrates the number of its occurrence.

Based on our network analysis, we identified 1,138 related articles on the genetic factors associated with arterial hypertension. Using VOSviewer software, we further divided these articles into 971 items, 19 clusters, 18,828 links, and a total link strength of 25,131. Our analysis revealed that the most frequent MeSH keywords related to genetic factors in arterial hypertension were genetic predisposition to disease, microRNA, gene expression regulation, and single nucleotide polymorphism (SNP), as shown in Table 1 and Figure 2. These keywords have been extensively studied in previous research on the genetic basis of arterial hypertension, suggesting their importance in understanding the underlying mechanisms of this disease.

**TABLE 1:** The most frequent MeSH keywords in the field of genetic factors and their roles in arterial hypertension (AH).

Keywords	Cluster	Link	Total link strength	Occurrence
Genetic predisposition to disease	12	342	930	107
MicroRNA	1	270	574	104
Gene expression regulation	3	357	645	88
Polymorphism, single nucleotide	2	263	666	87

**FIGURE 3:**
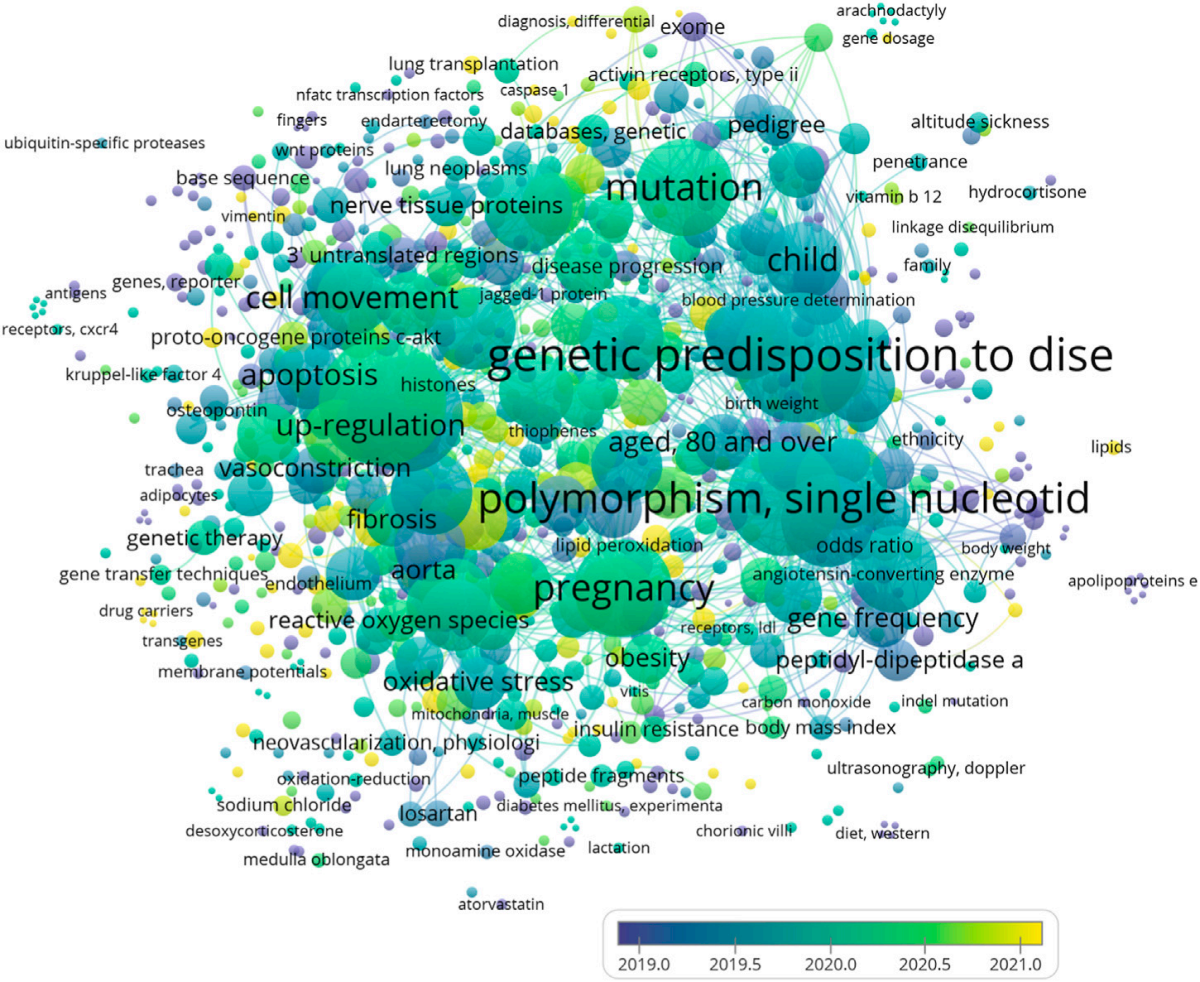
Overlay visualization of the most frequent Mesh keywords that are examined in studies in which the roles of genetic factors in arterial hypertension (AH) have been studied since 2018.

Besides, overlay visualization confirms that all the mentioned MeSH keywords have been examined 155 in related field since 2018 (Figure 3).

### 3.2 Genetic predisposition to disease and polymorphism, single nucleotide was two of the most studied MeSH keywords in studies in which the role of genetic factors in AH has studied

Various genes and their polymorphism and their different roles in AH are collected in Table 2. Table 2 provides information on genes, their polymorphisms, and alleles that play a role in arterial hypertension in various populations. The table lists the gene name, gene polymorphism, genotype, allele, the effect of the gene on arterial hypertension, ethnicity/territory, and reference. The table indicates that various genes such as 11β-HSD1, 5-HTT, 5-HTTLPR, ACE, ADD1, AGT, AGTR1, APOE, COX-2, DRD2, EDN1, ERBB3, HIF1α, IRS1, ITGA2, MMP-9, MTHFR, NOS2, RASA3, RNF213, SCNN1A, TBX2, TNF, TNFR2, and VDR have been identified to be associated with arterial hypertension in different populations. For each gene, the table provides details on the specific polymorphism and allele, along with the ethnicity/territory where the association was found and the reference for the study.

**TABLE 2:** Discovered genes, their normal functions, location, polymorphism and allele that play role in arterial hypertension (HTN) in various populations.

Gene name	Location	Activity of genes product	Gene polymorphism	Genotype	Allele	Effect of gene on arterial HTN	Ethnicity/Territory	References
11β-HSD1*	1q32.2	Conversion of the stress hormone cortisol to the inactive metabolite cortisone	rs45487298	A/A	A	Increase	Egyptian	Mo et al. (2022)
5-HTT	17q11.2	Transporting the neurotransmitter serotonin from synaptic spaces into presynaptic neurons	-	L/S	L	Increase	ND	Jiao et al. (2019), Villar et al. (2019)
5-HTTLPR	17q11.2	Transporting the neurotransmitter serotonin from synaptic spaces into presynaptic neurons	rs25531	S/S	-	Increase	Caucasian	Jaafar et al. (2018)
				S/S	-	Increase	Chinese	
				-	S	Increase	Germany	
				-	L	Increase	Malaysia	
ACE	17q23.3	Catalyzing the conversion of angiotensin I into a physiologically active peptide angiotensin II	-	D/D	D	Increase	Greek	Marushchak et al. (2019a), Ya et al. (2019)
				I/D	-		Ukrainian	
ADD1	4p16.3	Forming a substrate for protein kinases A and C	Gly460Trp	T/T	T	Increase	Ukrainian	Yermolenko et al. (2021)
				G/T		Increase		
AGT	1q42.2	Maintaining blood pressure, body fluid and electrolyte homeostasis	RS699	G/G	G	Increase	Arctic territory of Yakutia	Sofronova et al. (2019a), Marushchak et al. (2019b), Sofronova et al. (2019b)
			M235T	T/T	T	Increase	Ukrainian	
				M/T				
				G/G	G	Increase	Arctic territory of Yakutia	
AGTR1	3q24	Controlling blood pressure and volume in the cardiovascular system	A1166C	AC	-	Increase	Indonesian	Wulandari et al. (2018), Ou et al. (2022)
			rs4524238	G/G	G	Increase	Chinese	
APOE	19q13.32	Essential for the normal catabolism of triglyceride-rich lipoprotein constituents	rs7412	T/T		Increase	Chinese	Rao et al. (2022)
COX-2	1q25.2-q25.3.9	Transferring of electrons from cytochrome c to oxygen	rs5275	T/C	C	Increase	Turkish	Durmus et al. (2022)
			rs20417	G/C	C	Increase		
DRD2	11q23.2	Inhibiting adenylyl cyclase activity	−141c Ins/Del	D/D	D	Increase	Russian	Kosovtseva et al. (2018)
EDN1	6p24.1	Vasoconstriction	rs5370	-	-	Increase	ND	Jiao et al. (2019), Villar et al. (2019), Yang et al. (2021)
			rs397751713	A/A	A	Increase		
			LYS198ASN	Asn/Asn	Asn	Increase		
				Lys/Asn				
ERBB3	12q13.2	Kinase activity	rs705708	A/A	A	Decrease	Brazilian	Massignam et al. (2022)
HIF1α	14q23.2	Regulation of cellular and systemic homeostatic response to hypoxia	rs12434438	A/A	A	Increase	Japanese	Takagi et al. (2020)
IRS1	2q36.3	Phosphorylated by insulin receptor tyrosine kinase	rs2943640	C/C	C	Increase	Ukrainian	Vivsiana et al. (2021)
				C/A	-	Increase		
ITGA2	5q11.2	Mediating the adhesion of platelets and other cell types to the extracellular matrix	rs1126643	T/T	T	Increase	Russian	Shishkina et al. (2019)
				C/T				
MMP-9	20q13.12	Breakdown of extracellular matrix in normal physiological processes	rs11697325	A/A	A	Increase	Russian	Nikulina et al. (2022)
MTHFR	1p36.22	Catalyzing the conversion of 5,10-methylenetetrahydrofolate to 5-methyltetrahydrofolate	C677	T/T	T	Increase	Georgian	Garakanidze et al. (2018)
			C677T	T/T	T	Increase	Belarusian	Pavlova et al. (2018)
NOS2	17q11.2	Acting as a biologic mediator in several processes, including neurotransmission and antimicrobial and antitumoral activities	rs2779249	C/A	A	Increase	Siberian	Topchieva et al. (2019), Alyabyeva et al. (2023)
			rs2297518	G/A	A	Increase		
			rs1800482	C/C	C	Increase	Russian	
			rs3730017	T/T	T	Decrease		
RASA3	13q34	Stimulating the GTPase activity of Ras p21	rs9525228	E/A	-	Increase	ND	Prohaska et al. (2022)
RNF213	17q25.3	Involving in mediating protein-protein interactions	rs112735431	-	p.Arg4810Lys	Increase	Japanese	Suzuki et al. (2018)
SCNN1A	12p13.31	Controlling fluid and electrolyte transport across epithelia in many organs	rs11064153	T/T	T	Increase	Trans-Baikal	Pokoeva et al. (2022)
				C/C	C	Decrease		
TBX2	17q23.2	Involving in the regulation of developmental processes	rs8068318	-	-	Increase	ND	Abramova et al. (2020)
TNF	6p21.33	Involving in cell proliferation, differentiation, apoptosis, lipid metabolism, and coagulation *etc.*	G308A	G/A	A	Increase	Russian	Khlynova et al. (2020)
ТNFR2	1p36.22	Recruitment of two anti-apoptotic proteins, c-IAP1 and c-IAP2	rs1061624	A/A	A	Increase	Russian	Moskale et al. (2020)
VDR	12q13.11	Metabolism of minerals	FokI	f/f	f	Decrease	ND	Nunes et al. (2020)

*11β-HSD1, 11 beta hydroxysteroid dehydrogenase type 1; 5-HTT, serotonin transporter; 5-HTTLPR, Serotonin-transporter-linked promoter region; ACE, Angiotensin-I converting enzyme; ADD1, Alpha-Adducin 1; AGT, angiotensinogen; AGTR1, Angiotensinogen II, Type 1 Receptor; APOE, Apo lipoprotein E; COX2, Cytochrome c oxidase subunit 2; DRD2, D2-dopaminergic receptor; EDN1, Endothelin1; ERBB3, Erb-b2, receptor tyrosine kinase 3; HIF1α, Hypoxia-inducible factor 1α; IRS-1, Insulin receptor substrate-1; ITGA2, Integrin subunit alpha 2; MMP-9, Matrix metalloproteinase-9; MTHFR, methylenetetrahydrofolate reductase; NOS2, Nitric oxide synthase 2; RASA3, Ras GTPase, activating protein 3; RNF213, Ring finger protein 213; SCNN1A, Sodium channel epithelial 1 subunit alpha; TBX2, T-box transcription factor 2; TNF, tumor necrosis factor; ТNFR2, Tumor necrosis factor receptor 2; VDR, Vitamin D receptor.

### 3.3 Gene expression regulation was one of the most studied MeSH keywords in studies in which the role of genetic factors in AH has studied

Table 3 summarizes the epigenetic mechanisms that play a role in the development or worsening of arterial hypertension. It includes the gene name, the affecting mechanism(s) on the gene, and the result of the affecting mechanism(s) on the gene, along with the corresponding reference. The table shows that hyper methylation of the CBS promoter gene increases the risk of hypertension and stroke. Hyper-methylation of BMPR2 increases the risk of pulmonary arterial hypertension (PAH), while acetylation of H3K27 also increases the risk of PAH. Additionally, hypo methylation of SOCS3 promoter increases the risk of PAH. Other epigenetic factors and their functions in AH are displayed in Table 3.

**TABLE 3:** The epigenetic mechanisms that plays role in creating or deterioration of arterial hypertension (HTN) and pulmonary arterial hypertension (PAH).

Gene name	Affecting mechanism(s) on gene	Result of affecting mechanism(s) on gene	References
ABCA1	Hyper methylation	Decrease the risk of PAH	Napoli et al. (2019)
BMPR2	Hyper methylation	Increase the risk of PAH	Napoli et al. (2019), Bisserier et al. (2021)
		Decrease the risk of PAH	
CBS promoter	Hyper methylation	Increase the risk of HTN	Wang et al. (2019)
		Increase the risk of stroke	
CypA	Hyper acetylation	Increase the risk of PAH	Napoli et al. (2019)
H3K27	Acetylation	Increase the risk of PAH	Li et al. (2021a)
PGC-1α	Acetylation	Increase the risk of PAH	Napoli et al. (2019)
RASEF	Hyper methylation	Increase the risk of PAH	Li et al. (2019)
SOCS3 promoter	Hypo methylation	Increase the risk of PAH	Benincasa et al. (2022)

ABCA1, ATPbinding cassette 1; BMPR2, bone morphogenetic protein receptor type 2; CBS, promoter, cystathionine beta-synthase promoter; CypA, cyclophilin a; H3K27, histone H3 on lysine 27; PGC-1α, peroxisome proliferator-activated receptor-γ coactivator 1α; RASEF, RAS and EF-Hand domain containing; SOCS3, Suppressor of cytokine signaling 3.

### 3.4 MicroRNAs was one of the most studied MeSH keywords in studies in which the role of genetic factors in AH has studied

Table 4 contains different types of microRNAs that involve in AH and also their mechanism in mentioned disease. Table 4 presents various microRNAs that have been studied in the context of arterial hypertension (AH) from 2018 to 2023. These microRNAs have different roles in AH and their mechanisms are also mentioned in the table. miR-140-5p suppresses the proliferation, migration, and phenotypic variation of PASMCs. miR-204 suppresses Tgfbr2 while miR‐206 downregulates Kv1.5. miR-328-3p suppresses cell viability, migration, and the levels of VEGF, FGF-2 and HIF-1α in hypoxia-induced PASMCs. Other microRNAs have either amelioration or amplification roles in AH with different mechanisms of action. ND refers to “not determined”. The references for the studies are also mentioned in the table.

**TABLE 4:** Various microRNAs that they are involved in the process of arterial hypertension (AH) from 2018 to 2023.

microRNA	Role in AH	Mechanism(s)	References
miR-133a	Amelioration	ND	Koval et al. (2020), Yushko et al. (2020)
miR-140-5p	Amelioration	Suppressing the proliferation, migration, and phenotypic variation of PASMCs*	Zhu et al. (2019)
miR-144-3p	Amelioration	Downregulation of the expression of α-SMA, CTGF, and MYC in PAECs	Shi et al. (2022)
miR-150	Amelioration	ND	Russomanno et al. (2020)
miR-153	Amelioration	ND	Babicheva et al. (2020)
miR-15a-5p	Amplification	Reduce cell proliferation	Zhang et al. (2020)
		Increasing the levels of lactate dehydrogenase	
		Increasing the apoptosis of PASMCs	
		Increasing the activity of caspase-3/9	
		Increasing the protein expression of Bax in the PASMCs	
		Reduce the expression of Bcl-2 in the PASMCs	
miR-17	Amplification	ND	Li et al. (2020)
miR-204	Amelioration	Suppressing Tgfbr2	Yu et al. (2018)
miR‐206	Amplification	Downregulation of Kv1.5	Lv et al. (2019)
miR-20a	Amplification	ND	Li et al. (2020)
miR-221-3p	Amplification	Promoting pulmonary artery smooth muscle cells	Nie et al. (2019)
miR-30a	Amplification	Downregulation of P53 in PASMCs	Ma et al. (2021a)
miR-30d-5p	Amelioration	Attenuation of the PDGF-induced toxicity of PA-SMCs	Hu et al. (2022)
		Increasing the apoptosis of PASMCs	
		Downregulation of the expression levels of Notch-3	
miR-328-3p	Amelioration	Suppressing cell viability, migration, and the levels of VEGF, FGF-2 and HIF-1α in hypoxia-induced PASMCs	Yang et al. (2022)
miR-340-5p	Amelioration	Downregulation of the expression of IL-1β and IL-6	Ou et al. (2019)
miR-424 (322)	Amplification	Downregulation of SMURF1	Baptista et al. (2018)
miR-508-3p	Amplification	Increasing the proliferation and migration of PASMC	Ma et al. (2021b)
miR-663	Amelioration	Suppressing the PDGF-induced PASMCs proliferation and migration	Zhu et al. (2020), Li et al. (2021b)
		Downregulation of the expression and secretion of PDGF-induced TGF-β1	
miR-7110	Undetermined	ND	Johnson et al. (2020)

*CTGF, connective tissue growth factor; FGF-2, fibroblast growth factor 2; HIF, hypoxia inducible factor; IL, interleukin; Kv1.5, potassium voltage‐gated channel subfamily A member 5; MYC, myelocytomatosis; PASMC, pulmonary artery smooth muscle cell; PASMCs, pulmonary arterial smooth muscle cells; PDGF, platelet derived growth factor; SMA, smooth muscle actin; SMURF1, SMAD, ubiquitination regulatory factor 1; VEGF, vascular endothelial growth factor.

### 3.5 MicroRNAs with the most binding affinity to genes that involve in AH

After obtaining the structure of collectedd microRNAs from mirBASE (Kozomara and Griffiths-Jones, 2014) and also achieving the structure of target genes from the National Center for Biotechnology Information (NCBI) (Sayers et al., 2021), docking analysis of microRNAs and target genes was conducted by using RNAhybrid (Rehmsmeier et al., 2004).

**FIGURE 4:**
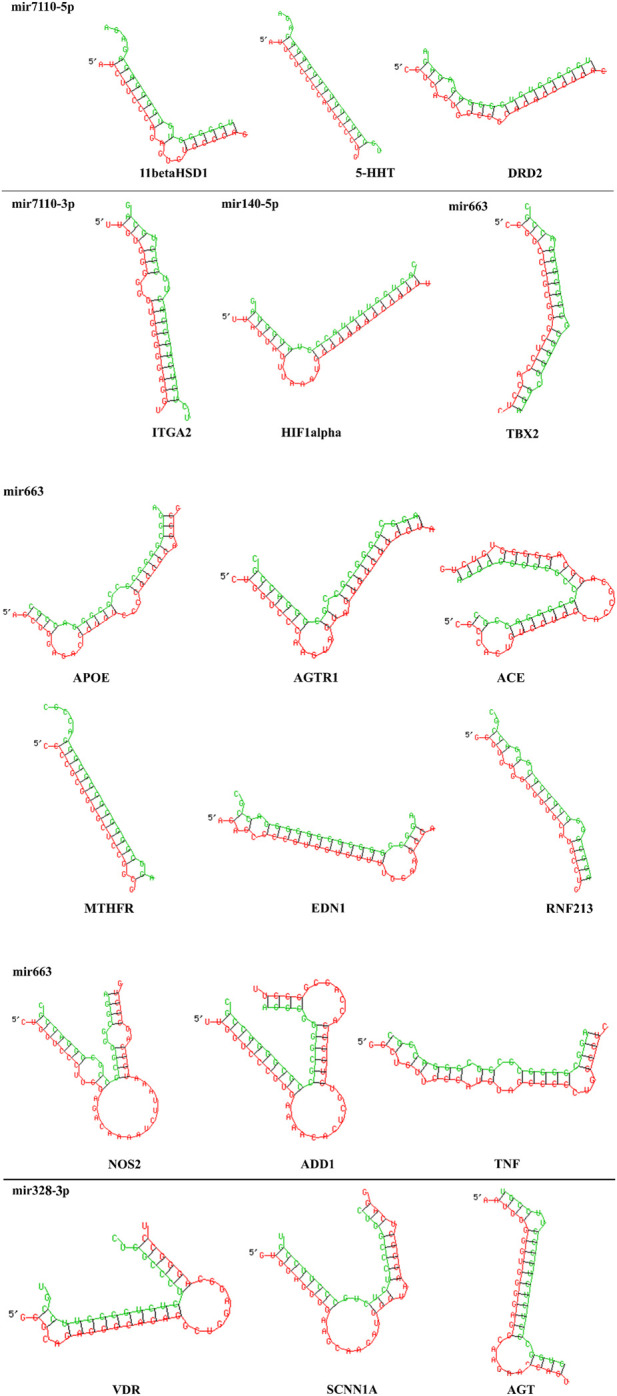
2D structure of binding site between MicroRNA-7110-5p and 11 beta hydroxysteroid dehydrogenase type 1 (11β-HSD1), serotonin transporter (5-HTT), and D2-dopaminergic receptor (DRD2). Moreover, MiR-7110-3p has the most binding affinity to integrin subunit alpha 2I (ITGA2) and MicroRNA-663 and Angiotensinogen II Type 1 Receptor (AGTR1), Angiotensin-converting enzyme (ACE), and methylenetetrahydrofolate reductase (MTHFR), Apo lipoprotein E (APOE), Angiotensinogen II Type 1 Receptor (AGTR1), Angiotensin-converting enzyme (ACE), methylenetetrahydrofolate reductase (MTHFR), endothelin1 (EDN1) ring finger protein 213 (RNF213), Alpha-Adducin 1 (ADD1), and tumor necrosis factor (TNF), MiR-328-3p and vitamin D receptor (VDR), sodium channel epithelial 1 subunit alpha (SCNN1A) and angiotensinogen (AGT), MiR-140-5p and Hypoxia-inducible factor 1α (HIF1α). Each microRNA with its target genes are displayed and they are separated by a horizontal line. Red chains and green chains represent target gene and microRNA, respectively.

Table 5 has the detailed information about binding affinity of various microRNA and genes that play a part in AH. According to remarked table, MicroRNA-7110-5p has the most binding affinity to 11 beta hydroxysteroid dehydrogenase type 1 (11β-HSD1), serotonin transporter (5-HTT), and D2-dopaminergic receptor (DRD2). Moreover, MiR-7110-3p has the most binding affinity to integrin subunit alpha 2I (ITGA2) and MicroRNA-663. Besides, MicroRNA-663 demonstrated the most binding affinity to Angiotensinogen II Type 1 Receptor (AGTR1), Angiotensin-converting enzyme (ACE), and methylenetetrahydrofolate reductase (MTHFR), Apo lipoprotein E (APOE), Angiotensinogen II Type 1 Receptor (AGTR1), Angiotensin-converting enzyme (ACE), methylenetetrahydrofolate reductase (MTHFR), endothelin1 (EDN1) ring finger protein 213 (RNF213), Alpha-Adducin 1 (ADD1), and tumor necrosis factor (TNF). Furthermore, MiR-328-3p has the most binding affinity to vitamin D receptor (VDR), sodium channel epithelial 1 subunit alpha (SCNN1A) and angiotensinogen (AGT), and ultimately, MiR-140-5p has the most binding affinity to Hypoxia-inducible factor 1α (HIF1α). Besides, Figure 4 demonstrated the nucleotides that involve in the binding site of all mentioned microRNAs and genes.

**TABLE 5:** The binding affinity (Kcal/mole) of microRNAs and genes that play a part in arterial hypertension (AH).

MicroRNAs	Genes																	
	APOE	AGTR1	11β-HSD1	ACE	MTHFR	5-HHT	EDN1	VDR	RNF213	NOS2	HIF1A	ADD1	TNF	SCNN1A	AGT	TBX2	ITGA2	DRD2
miR-7110-5p	−29.9	−24.6	−30.4	−32.6	−36	−34.4	−26	−34.7	−32	−30.3	−21.7	−33	−30.4	−31.7	−29.8	−31.5	−34.9	−34.6
miR-7110-3p	−22.5	−22.2	−25.6	−30.7	−34.3	−29	−25.5	−31.5	−27.4	−28	−16.5	−29	−23.9	−26.7	−28.11	−37.6	−35.5	−34.3
miR-140-5p	−23.2	−19.6	−19.1	−25.4	−27	−24.5	−21.4	−27.5	−25	−24.3	−26.1	−24.2	−23.4	−22.1	−21.8	−24.9	−22.9	−23.5
miR-221-5p	−14.6	−18.5	−16.3	−21.9	−22.4	−22.1	−18.3	−20.4	−22.4	−21.6	−19.8	−20.8	−18.7	−20.6	−20.1	−27.4	−24.6	−19.8
miR-17-5p	−20.6	−25.3	−21.5	−27.1	−28.8	−28.5	−22.1	−24.9	−22.8	−23.1	−22.3	−24.2	−29	−23	−24.2	−26.3	−28.1	−24.5
miR-20a-5p	−20.5	−24	−19.9	−24.2	−27.1	−26.9	−20.1	−23.3	−23.2	−22.4	−21.4	−23.1	−27.5	−22.3	−23.7	−24.4	−25.1	−23
miR-328-3p	−19.3	−24.4	−26.6	−31.5	−37.5	−31.5	−27	−36.1	−28	−28.8	−17.6	−35.9	−28.7	−32.2	−30.3	−35.1	−33.1	−32.4
miR-424-5p	−14.5	−20.3	−17.8	−22.3	−21.6	−23.7	−19.1	−23.8	−19	−26.9	−18.8	−26.5	−19.6	−21.4	−18.6	−24.3	−23.6	−20.4
miR-204-5p	−18.1	−17.6	−27.3	−22.9	−30.4	−27.1	−22.9	−26.2	−26.7	−26.9	−19.5	−23.8	−21.4	−22.7	−24.5	−31	−28.6	−28.4
miR-133a-3p	−22.1	−17.1	−27.8	−26.2	−30.2	−29.1	−24.3	−28.7	−27.4	−22.6	−17.1	−33.6	−25.2	−26.1	−23.3	−28.1	−27.6	−27.5
miR-133a-5p	−16.6	−19.3	−17.8	−26.4	−23.6	−21.2	−20.4	−20.6	−23.9	−20.8	−23.6	−24.9	−23.3	−20.6	−22.2	−22.1	−25.3	−23
miR-15a-5p	−17.3	−23.1	−17	−24	−22.9	−24.6	−20.5	−23.9	−21.9	−21.5	−20	−24.4	−20.5	−22	−27.3	−25.1	−23	−21.7
miR-508-3p	−19.7	−24.8	−23.9	−24.2	−29.1	−29.3	−26.7	−26.1	−24.4	−21	−25.2	−28.4	−24.4	−25.8	−20.2	−24.2	−23.7	−24.3
miR-663	−36.6	−28.2	−24.3	−35.2	−39.7	−32.8	−31.2	−35.2	−32.8	−32.6	−25.1	−37.7	−32	−31.7	−29.8	−39	−33.6	−33.7
miR-30a-5p	−19.7	−18.4	−18.4	−20.2	−22.5	−21.9	−23.1	−21.7	−20.9	−19.1	−22.4	−21.5	−19.4	−21.1	−20.8	−25.1	−23.7	−21
miR-150-5p	−17.6	−17.3	−28.4	−24.1	−27.6	−23.4	−23	−30.7	−28.2	−27.9	−16.7	−26.7	−29.6	−27.3	−30.2	−32.3	−28.9	−31.7
miR-30d-5p	−20.9	−17.8	−16.5	−22.2	−23.5	−22.2	−26.1	−24.1	−21.8	−19.6	−21.3	−21	−20	−23.2	−23.8	−24.2	−24.7	−21.5
miR-144-3p	−10.5	−19.7	−14.2	−17	−21.2	−20.4	−16.6	−20.9	−16.2	−20.5	−18.3	−18.5	−15.9	−18.5	−15.7	−23.6	−21.8	−15.7
miR-206	−20.7	−22.8	−19.1	−25.3	−24.7	−32.5	−24.9	−22.9	−24.4	−22	−20.7	−25.2	−21.3	−23.8	−21.5	−20.2	−23.8	−24.7
miR-340-5p	−13.9	−17.8	−19.1	−16.9	−19	−19.2	−17.1	−21.4	−19.9	−16.9	−18.6	−19.3	−20.6	−16.6	−17.8	−18.7	−18.7	−17.8
miR-153-5p	−18.6	−24.9	−23	−24.3	−24	−27.3	−20.8	−21.6	−20.8	−23.3	−23.6	−25.4	−20.6	−19.7	−24.4	−24.7	−23.4	−23.1
miR-153-3p	−12.2	−21.2	−16.1	−20.4	−20.9	−21.7	−17.1	−18.3	−20	−18.4	−18.4	−19.8	−18.5	−22.1	−17.8	−18	−21.9	−18.8

## 4 Discussion

### 4.1 Genetic predisposition to disease and polymorphism, single nucleotide was two of the most studied MeSH keywords in studies

Previous studies have confirmed the role of various genes and their polymorphism and also their single nucleotide in the pathophysiology of AH (Shnayder et al., 2021). Our study demonstrated that Genetic predisposition to disease and Polymorphism, single nucleotide are two factors that have been most frequent MeSH key words in related studies from 2018 to 2023 (Table 1; Figure 2). This finding is confirmed by prior surveys in which the role of genetic factors has been examined. Besides, previous surveys have mentioned that scientists have been trying to discover various genetic factors and pathways that involve in pathophysiology of AH (Friso et al., 2015).

### 4.2 Gene expression regulation

One of the most interesting keywords in the field of the genetic factors and their role in AH is gene expression regulation (Table 1). On the other side, one important way in order to regulate gene expression is using epigenetic factors for regulating gene expression. In better words, epigenetic factors, have gained the attention of scientists for examining their role in AH (Friso et al., 2015). Epigenetic factors are defined as heritable traits that alter the expression of human genes without making any change in the structure of DNA sequence. They have involved in the pathogenesis of many cardiovascular diseases including PAH. In other words, epigenetic factors in accompanying with genetic mechanisms play a crucial role in the dysregulation of network-based molecular architecture in cellular level in AH (Napoli et al., 2019). DNA methylation, histone modifications, mRNA methylation, and noncoding RNA modifications are major types of epigenetic factors (Barroso et al., 2014). These epigenetic mechanisms with genetic factors and environmental risk factors play a crucial role in occurrence and intensity of both vascular endothelial cells as well as AH (Napoli et al., 2019). Besides, based on previous researches, there are five available curative strategies in order to treat AH including endothelin receptor antagonists, prostanoid analogues, phosphodiesterase type 5 inhibitors, nonprostanoid IP receptor agonists, and soluble guanylyl cyclase stimulators. Albeit the benefits of all mentioned curative strategies, some serious side effects still exists (Jain et al., 2017). Hence, scientists have tried to develop more effective therapeutic agents. One of these curative agents are epigenetic drugs (epidrugs) which have gained the attention of researchers in recent years (Napoli et al., 2016). For example, SIN3a (switch-independent 3a), a transcriptional regulator has been utilized to regulate methylation and expression levels of the bone morphogenetic protein receptor type 2 (BMPR2) gene in human pulmonary arterial smooth muscle cells. SIN3a suppresses the methylation of BMPR2 and increased the expression of it. Thus, SIN3a can protect these cells against pathological changes that end to AH (Bisserier et al., 2021). Furthermore, previous studies demonstrated that hyper methylation of BMPR2 can oppress the proliferation of vascular cells which is crucial for developing and intensifying PAH. This finding make BMPR2 a proper candidate for being an effective epidrug and also a target for anti-AH therapeutic agents in near future (Napoli et al., 2019). In fact, SIN3a exerted its action by downregulating the expression of DNMT1 (DNA methyltransferase 1) and EZH2 (enhancer of zeste 2 polycomb repressive complex 2) and up-regulating the expression of the TET1 (ten-eleven translocation methylcytosine dioxygenase 1) (Bisserier et al., 2021). The other fascinating epigenetic factor that seems to be a potential future epidrug is hyper methylated ABCA1 (ATPbinding cassette 1). This microRNA blocks vascular cells in mitotic phase and prevent AH to become progressive (Napoli et al., 2019).

Notably, oxidative stress underlying PAH phenotype is formed by cooperation between hyper acetylation of cyclophilin a (CypA) with downregulation of antioxidant superoxide dismutase 3 (SOD3) (Napoli et al., 2019). On the other side, scientists have found out that some epigenetic modifications can worsen AH. For instance, the acetylation of H3K27ac (histone H3 lysine 27) by acetyl coenzyme A can create proper condition for the acetyltransferase KAT2B (lysine acetyltransferase 2B) and activation of genes that involve in cell cycle and metabolic events, that is, required for aldehyde dehydrogenase family 1 member 3 (ALDH1A3) - dependent proliferation and glycolysis. Finally, this activation leads to amplification of AH (Li D. et al., 2021) which we have displayed in Table 3.

Moreover, acetylation of peroxisome proliferator-activated receptor-γ coactivator 1α (PGC-1α) causes sustaining the vascular proliferation in the condition of PAH through downregulation of SIRT1 HDAC (histone deacetylase) (Napoli et al., 2019).

Besides, scientists have found that there is a connection between pulmonary arterial hypertension (PAH) in rat model and hyper methylation of RASEF. In this survey, the exposure of rats to smoking caused increase the level of hyper methylation in RASEF and occurrence of some changes including right ventricular hypertrophy, increasing the thickness right ventricular, and raising systolic blood pressure was observed. Notably, remarked research have conveyed that overespression of RASEF can inhibit AH through downregulation of phospho-AKT (Ser473), proliferating cell nuclear antigen (PCNA), and matrix metalloproteinase 9 (MMP9). Thereby, it is plausible that hyper methylation of RASEF exerts its role through upregulation of mentioned factors. Ultimately, this study endorsed the companionship between hyper methylation of RASEF and PAH (Li et al., 2019).

In brief, according to Table 3, DNA hyper methylation, hyper acetylation and DNA acetylation have been introduced as epigenetic factors in order to regulate gene expression in AH since 2018. As you can see in remarked table, epigenetic factors have a variety of effects in the process of pathology of AH. Interestingly, prior researches have demonstrated that post-translational modifications including hyper methylation and acetylation are known as a crucial factor for developing AH and also have diverse impacts on mentioned pathological condition (Friso et al., 2015). Thus, the results from our bibliometric analysis are confirmed by previous surveys.

### 4.3 MicroRNAs was one of the most studied MeSH keywords in studies in which the role of genetic factors in AH has studied

**FIGURE 5:**
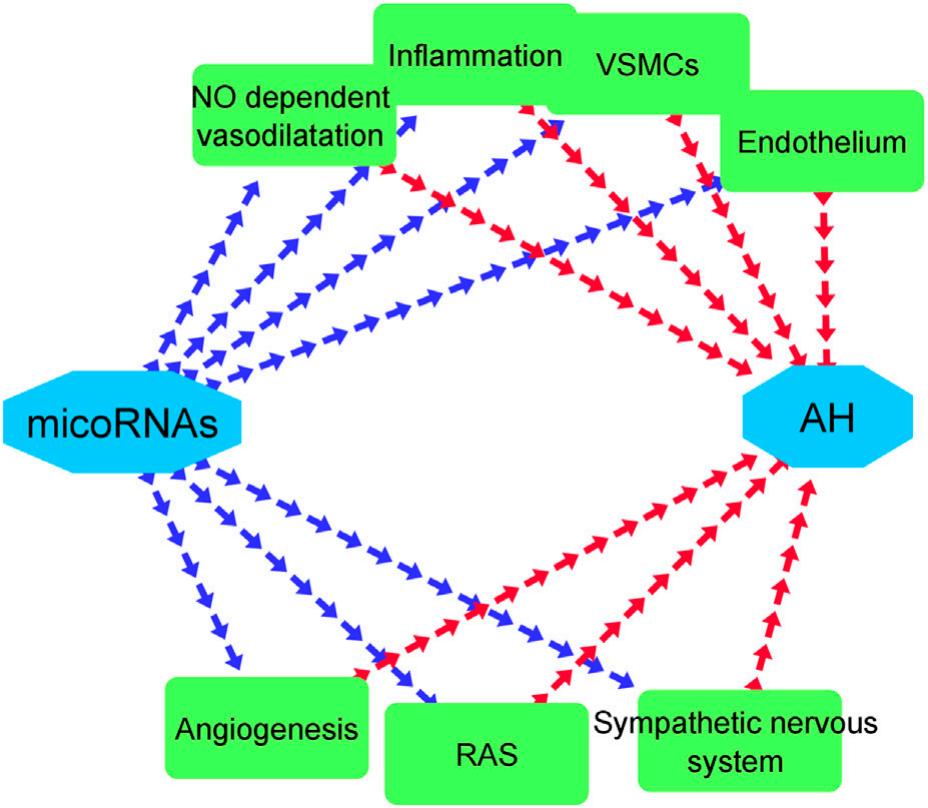
Various effects of microRNAs in the pathogenesis of AH. Blue and red arrows represent the effects of microRNAs on mechanisms that involve in AH and the impacts of these mechanisms on AH, respectively.

MicroRNAs (miRNAs) are small non-coding RNA molecules that previous surveys have proved that they are expressed in AH and they can play a crucial part in the pathogenesis of AH (Synetos et al., 2013). These kinds of RNAs involve in various mechanisms that ends to effect on AH including participating in the functions of arterial endothelial cells which contain receiving mechanical stimuli and transform them into intracellular signals, inducing alteration of cellular structure and function. The other role of microRNAs in the pathophysiology of AH is playing a remarkable role in regulation of leucocyte adhesion on the surface of endothelial cells, inflammatory processes, vasodilatory activities, and proliferation of endothelium. Besides, microRNAs can effect Nitric oxide (NO) dependent vasodilatation through adjusting the level of NO in human body, since upregulation of NO can lead to increase in blood pressure. The other impact of microRNA is involving in the regulation of the activity of vascular smooth muscle cells (VSMCs) (Klimczak et al., 2017). VSMCs have the capability for adapting their phenotype based on current condition. In other words, when these cells were undergone vascular damage caused by high blood pressure, they turn from contractile cells to synthetic ones (Owens et al., 2004). Subsequently, they start to raise proliferation, migration, collagen and extracellular matrix synthesis, as well as deduct expression of contractility markers (Kawai-Kowase and Owens, 2007). Interestingly, microRNAs have the capability to adjust phenotype of VMSCs in the situations in which vascular damages are occurred by AH (Klimczak et al., 2017). As we mentioned in Table 4, some microRNAs including miR-15a-5p, miR-30a, miR-30d-5p, miR-328-3p, and miR-663 are responsible for making VSMCs to form proper phenotype during damaging process of AH. Furthermore, microRNAs contribute to the process of AH by interfering in the functions of sympathetic nervous system and renin–angiotensin system (RAS) (Klimczak et al., 2017). Although the studies about the roles of microRNAs in the sympathetic nervous system have been limited but some surveys have demonstrated these RNAs can link sympathetic nervous system with RAS and also have the ability to regulate renin mRNA (Jackson et al., 2013). On the other side, RAS which plays a vital role in the pathogenesis of AH, has the capability to effect various organs both directly and indirectly can modulate by 55 microRNA species through activation of angiotensin II type 1 receptor (AT1R) (Velloso et al., 1996; Kemp et al., 2014). Ultimately, another function of microRNAs in the process of pathogenesis of AH in inhibiting inflammation by downregulation of IL6 (Wang et al., 2013). Figure 5 displays a whole paradigm of the various roles of microRNAs in the pathology of AH. Interestingly, our Network visualization displayed that a major number of early studies in the field of examining the genetic functions in AH have focused on miRNAs. Besides, some prior surveys have tried to utilize *in silico* studies in order to discover the role of miRNAs and their probable target genes that play a crucial role in AH. For example, in early studies, the role of circulating Let-7b on pulmonary hypertension was examined and its target genes were determined through *in silico* analysis (Guo et al., 2014). Moreover, other *in silico* surveys have demonstrated the effects of some gene polymorphisms that play a part in AH on the function of some miRNAs (Ya et al., 2015). Our study demonstrated that miRNAs can be an important element in the process of pathophysiology of AH. This is in line with other previous similar studies. On the other side, Table 4 demonstrates various classes of miRNAs and their detailed role in AH. As it is displayed in this table, miRNAs have various effects in AH. This result in line with the findings of prior surveys (Synetos et al., 2013).

### 4.4 Overlay visualization of the most frequent MeSH keywords in related field

According to our bibliometric analysis, all of the most frequent MeSH keywords in related field that are listed in Table 1 have been undergone various studies since 2019 (Figure 3). Besides, all of them have gained the attention of scientists that have vast activities in the field of AH. Thus, the result of overlay visualization in present survey confirms that scientists have been trying to elucidate the role of various genetic factors in the pathophysiology of AH (Figure 3).

### 4.5 MicroRNAs with the most binding affinity to genes that involve in AH

#### 4.5.1 MicroRNA-7110-5p has the most binding affinity to 11 beta hydroxysteroid dehydrogenase type 1 (11β-HSD1)

Prior studies have confirmed the role of microRNA-7110-5p in the patients with AH (Johnson et al., 2020). The exact role of this MicroRNA has not been revealed yet but early surveys have endorsed the existence of MicroRNA-7110-5p in patients with AH (Johnson et al., 2020). Moreover, our *in silico* examinations display the high binding affinity of mentioned microRNA to 11β-HSD1. The product of this gene is 11β-HSD1enzyme and this enzyme in accompanying with 11β-hydroxysteroid dehydrogenase type 2 (11βHSD2) enzyme involve in cortisol regulation. The cortisol regulation is a vital factor in the adjustment of arterial hypertension because the excessive amount of cortisol is associated with HTN (Mo et al., 2022). This means that 11β-HSD1 worsens the condition of AH (Mo et al., 2022). Hence, this *in silico* finding shows that maybe the effects of microRNA-7110-5p on AH are exerted by its effect on remarked gene. Despite of this finding, more *in-vitro* and *in-vivo* studies are necessary to confirm mentioned *in silico* result.

#### 4.5.2 MicroRNA-7110-5p has the most binding affinity to serotonin transporter (5-HTT) and D2-dopaminergic receptor (DRD2)

Serotonin transporter (5-HTT) gene in connected with 5-hydroxytryptamine and its transporter. These two mentioned factor contribute to the hyperplasia of vascular smooth muscle and also vascular remodeling associated with PAH. Despite of that, the role of 5-HTT in some subtypes of PAH and also some ethnicities is still unknown (Jiao et al., 2019). But the stimulatory effects of 5-HTT on AH has been clarified by previous studies (Table 2).

Besides, a survey on 110 men from in the age range of 14–17-year old of Caucasian patients with EH demonstrated that the association of polymorphism −141C 1/D D2-dopaminergic receptor (DRD2) with the occurrence and intensity of AH (Kosovtseva et al., 2018). Briefly, prior researches have demonstrated that both 5-HTT and DRD2 can increase the possibility of occurrence of AH (Table 2). Moreover, our present work displayed the high affinity of microRNA-7110-5p to both mentioned receptor (Table 5). Thus, regarding to the uncertain effect of mentioned microRNA in the process of pathology of AH, this hypothesis is mentioned that microRNA-7110-5p may exert its effects on AH through 5-HTT and DRD2. Notably, more researches are highly necessary in order to prove this theory.

#### 4.5.3 MiR-7110-3p has the most binding affinity to integrin subunit alpha 2I (ITGA2)

Genotyping of ITGA2 in 47 patients with AH demonstrated that the prevalence of C/C, C/T, and T/T genotypes of ITGA2 gene among hypertensive patients was 38.3%, 48.9%, and 12.8%, respectively (Shishkina et al., 2019). This survey have also displayed that upregulation of ITGA2 can lead to increases in the amount of the risk of AH (Table 2). Besides, *in silico* analysis revealed the high tendency of mentioned microRNA to ITGA2. Therefore, this *in silico* finding may reveal some aspects of the role of MiR-7110-3p in the process of pathology of AH.

#### 4.5.4 MicroRNA-663 has the most binding affinity to angiotensinogen II type 1 receptor (AGTR1)

Angiotensinogen II Type 1 Receptor (AGTR1) is well-known as predisposition factor of AH in Asian and Caucasian population. Moreover, AGTR1 plays an important role in Renin-Angiotensin-Aldosteron-System (RAAS). Some important functions of AGTR1 are mediation of the classical biological actions of angiotensin, activation of a phosphatidylinositol-calcium second messenger system, involving in natriuretic hormone function, sodium absorption dysfunctions, thickening of arterial walls, renal sodium reabsorption and retention. Furthermore, the expression of AGTR1 ends in the raise of the level of SBP (systolic blood pressure) and DBP (diastolic blood pressure) (Wulandari et al., 2018).

This receptor was undergone *in silico* analysis and it was shown that microRNA miR-663a demonstrated a high affinity to this gene (Table 5). This microRNA has demonstrated ameliorative effect on AH before (Table 4). In fact, miR-663a participates in the process of inhibiting the vascular remodeling in normal pulmonary artery cells and oppressing the creation of pulmonary artery hypertension by inhibiting transforming growth factor-beta (TGF-β) (Li P. et al., 2021). In conclusion, this *in silico* finding can be confirmed by previous studies.

#### 4.5.5 MicroRNA-663 has the most binding affinity to angiotensin-converting enzyme (ACE) and methylenetetrahydrofolate reductase (MTHFR)

MicroRNA-663 demonstrated the highest affinity to ACE and Methylenetetrahydrofolate reductase (MTHFR) (Table 5). As mentioned before, this microRNA has exerted ameliorative effects on AH through inhibition of TGF-β, vascular remodeling in pulmonary arteries, ventricular hypertrophy, inhibition of PDGF-BB-induced PAMSCs proliferation, and also suppression on TGF-β1/smad2/3 signaling pathway (Table 4) (Zhu et al., 2020; Li P. et al., 2021). On the contrary, ACE and MTHFR gene increases the risk of AH (Table 2). The association of high blood pressure in two patients with the D variant of the angiotensin-converting enzyme (ACE) has been displayed before (Ya et al., 2019). Moreover, in a survey performed in 2019, 96 patients were undergone a research and it was demonstrated that the existence of D allele of the ACE gene may raise the risk for AH in patients with chronic obstructive pulmonary disease (COPD) (Marushchak et al., 2019a). Notably, MTHFR based hyperhomocysteinemia (HHcy) has shown a synergic effect with AH which shows that MTHFR can be used as a useful marker in order to be a predictive factor for AH (Wang et al., 2015).

Thus, our *in silico* result about the affinity of mentioned microRNA to ACE and MTHFR represents the possibility of exerting the ameliorative effects of microRNA-663 by inhibiting the ACE gene but the necessity of more researches for proving this result is still exists.

#### 4.5.6 MiR-328-3p has the most binding affinity to vitamin D receptor (VDR), sodium channel epithelial 1 subunit alpha (SCNN1A) and angiotensinogen (AGT)

The association between FokI polymorphism in the vitamin D receptor (VDR) gene and susceptibility to AH is still under debate but FokI, a functional polymorphism of VDR, has had a remarkable correlation with the incidence of AH. Allele f of the VDR gene with remarked polymorphism was found to be associated with a remarkably lower occurrence of HT (Nunes et al., 2020).

SCNN1A has also undergone a survey in which the role of the rs11064153 variant of this gene in occurrence of AH was studied and carriage of the T/T genotype of the SCNN1A gene increased the likelihood of AH in patients. On the contrary, carrying allele C and the C/C SCNN1A genotype deducted the possibility of developing AH (Pokoeva et al., 2022).

Early surveys have also displayed that homozygous GG genotype for the AGT SNP rs699 is correlated with high AH especially with high levels of SBP (Sofronova SI. et al., 2019). In another study, the presence of Т allele of the AGT gene with increase the risk of AH in patients suffer from COPD (Marushchak et al., 2019b).

MiR-328-3p is known as a potential target of long noncoding RNA, LINC00963. This microRNA inhibited by remarked molecule and this inhibition results is intensifying of AH because the activation of MiR-328-3p ends in downregulation of the levels of Vascular endothelial growth factor (VEGF), Fibroblast Growth Factor 2 (FGF-2) and Hypoxia-inducible factor 1-alpha (HIF-1α) in hypoxia-induced human pulmonary artery smooth muscle cells (PASMCs) (Yang et al., 2022).

In brief, the anti-AH effects of VDR and SCNN1A has been proved by prior researches before (Table 2). Besides, microRNA-328-3p showed the most binding affinity to these genes. Moreover, the inhibitory effects of mentioned microRNA on AH have been displayed before (Table 4). Thus, the *in silico* finding is in line with the anti-AH results of previous studies and it may show the probable anti-AH mechanism of remarked microRNA. AGT also increases the risk of AH (Table 2). The high affinity of microRNA-328-3p may represent that anti-AH influences of this microRNA may be exerted by its inhibitory tendency to remarked gene. On the other hand, more surveys are needed to validate mentioned *in silico* findings.

#### 4.5.7 MiR-663 has the most binding affinity to other receptors that worsen AH

Apo lipoprotein E (APOE) (Rao et al., 2022), Angiotensinogen II Type 1 Receptor (AGTR1) (Wulandari et al., 2018), Angiotensin-converting enzyme (ACE) (Ya et al., 2019), methylenetetrahydrofolate reductase (MTHFR) (Garakanidze et al., 2018), endothelin1 (EDN1) (Yang et al., 2021), ring finger protein 213 (RNF213) (Suzuki et al., 2018), Alpha-Adducin 1 (ADD1) (Yermolenko et al., 2021), tumor necrosis factor (TNF) (Khlynova et al., 2020) and T-box transcription factor 2 (TBX2) (Abramova et al., 2020) involve in the AH process and the overexpression of them can cause amplification in AH.

Despite this, microRNA-663 attenuates the process of AH (Table 4). Moreover, our *in silico* finding about this microRNA and its high affinity to mentioned genes remark that this microRNA can exert its anti-AH activities through the inhibition of mentioned genes but more investigations are essential to prove this result.

#### 4.5.8 MiR-663 has the most binding affinity to nitric oxide synthase 2 (NOS2)

MiR-663 demonstrated the most tendency to NOS2 which ameliorates AH. on the other side, early studies have shown that this microRNA decreases arterial blood pressure in patients as this microRNA has exerted suppressive effects on AH by inhibition of TGF-β and subsequently, oppression of TGF-β1/smad2/3 signaling, vascular remodeling (Zhu et al., 2020; Li et al., 2021). Furthermore, in a study performed on 91 Caucasian participants from Eastern Siberia, it was found out that rs2779249 and rs229718 polymorphism of the NOS2 gene were associated with AH (Alyabyeva et al., 2023). Thus, our mentioned *in silico* finding and the results of previous studies have shown the possibility of inhibitory effect of microRNA-663 on NOS2 in AH condition. Although, more examinations are highly essential in order to display and confirm this *in silico* result.

#### 4.5.9 MiR-140-5p has the most binding affinity to hypoxia-inducible factor 1α (HIF1α)

Under hypoxic situation, HIF1α is produced by endothelial cells and it is associated with AH. The expression of the AA genotype at rs12434438 in gene is in accompanying with intensity of AH (Takagi et al., 2020).

Moreover, PAH is one of significant sorts of AH and the major features of PAH are hyper proliferation of PASMCs and apoptosis resistance. Therefore, in previous researches, it was proved that overexpression of miR‐140‐5p inhibits pathogenesis of PAH through suppressing of the proliferation, migration, and phenotypic variation of PASMCs (Zhu et al., 2019).

Briefly, MiR-140-5p have had ameliorative influences on AH (Table 4). This microRNA demonstrated the most tendency to HIF1α (Table 5). This gene raises the amount of arterial hypertension and worsen it (Table 2). Thus, we can claim that according to previous surveys and our *in silico* finding, this microRNA may exert its inhibitory action through the suppression of mentioned gene. Hence, more examinations are necessary to validate this finding.

### 4.6 Final network of the effect of MicroRNA with the most binding affinity to genes that involve in AH

**FIGURE 6:**
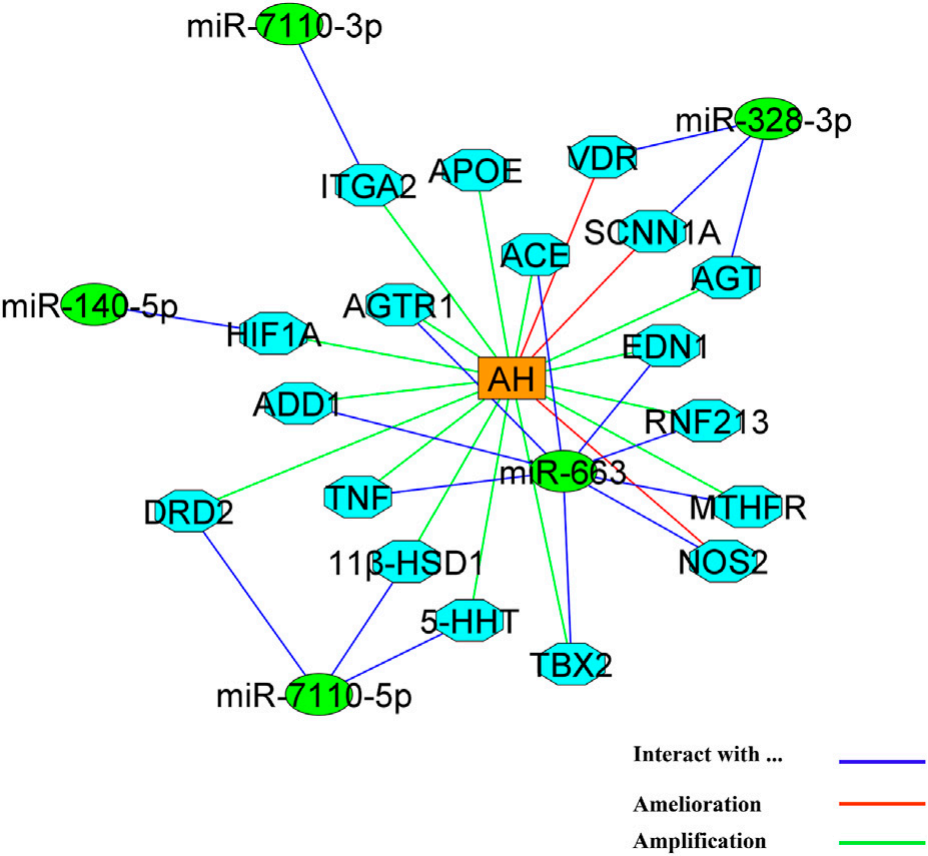
The network of action of microRNAs with the most tendency to genes that play a role in AH. MicroRNAs and genes are represented by green circles and light blue octagons, respectively. AH is located as an orange triangle in the center of figure.

MicroRNAs have an important role in the process of AH. these molecules have gained the attention of scientists who works on AH (Table 1 and Figure 2). Despite this, the exact and detailed mechanisms of these molecules in pathophysiology of AH is still unclear and needs various *in-vitro* and *in-vivo* examinations (Johnson et al., 2020). Hence, in this study, we tried to elucidate a part of the role of recent discovered microRNAs that have shown remarkable impacts in the pathophysiology of AH in *in silico* condition (Table 5). Moreover, we demonstrated the possible network of the role of microRNAs with the most affinity to discovered genes that play a part in AH in Figure 6. Although the findings of present study may guide researchers to pay more attention to mentioned microRNAs and genes but more surveys need to be conducted in the future for revealing the detailed mechanisms of action of microRNAs in AH.

## 5 Conclusion

According to our findings, genetic factors that involve in AH have been one of the most attractive fields that are studied by scientists in recent years. Besides, epigenetic factors, which have demonstrated different roles in pathology of AH, have attracted the attention of scientists as a novel therapeutic strategy in order to cure AH. Epigenetic alterations in some genes including ABCA1, BMPR2, CypA, H3K27, PGC-1α, RASEF, and SOCS3 hhave had some remarkable effect on AH and these genes can be a potential target to manufacture effective epidrugs.

Moreover, present survey demonstrated that microRNAs plays a crucial role in the pathophysiology of AH and they show their *in silico* potential in order to play an important role in ameliorating and amplification of AH. On the other side, miR-7110-5p, miR-7110-3p, miR-663, miR-328-3p, and miR-140-5p has the most binding affinity to genes that are involve in AH and they may be used as precious agents for diagnosing and treating AH in future.

## References

[B1] Abramova M. Y. Usacheva T. A. Sorokina I. N. Verzilina I. N. Polyakova I. S. Kulikovsky V. F. (2020). Insilico test of functional role of rs8068318 polymorphism of arterial hypertension-associated TBX2 candidate gene. Sys Rev. Pharm. 11 (6), 07–10. 10.31838/srp.2020.6.02

[B2] Alyabyeva P. V. Petrova M. M. Dmitrenko D. V. Garganeeva N. P. Chumakova G. A. Al-Zamil M. (2023). Association of single-nucleotide polymorphisms Rs2779249 (chr17: 26128581 C> A) and Rs rs2297518 (chr17: chr17: 27769571 G> A) of the NOS2 gene with tension-type headache and arterial hypertension overlap syndrome in Eastern Siberia. Genes 14 (2), 513. 10.3390/genes14020513 36833440 PMC9957272

[B4] Aryal B. Suárez Y. (2019). Non-coding RNA regulation of endothelial and macrophage functions during atherosclerosis. Vasc. Pharmacol. 114, 64–75. 10.1016/j.vph.2018.03.001 PMC6177333 29551552

[B5] Babicheva A. Jain P. Zhao T. Xiong M. Lai N. Makino A. (2020). Decreased microRNA‐153 promotes endothelial-to-mesenchymal transition in idiopathic pulmonary arterial hypertension. FASEB J. 34 (S1), 1. 10.1096/fasebj.2020.34.s1.07429

[B6] Baptista R. Marques C. Catarino S. Enguita F. J. Costa M. C. Matafome P. (2018). MicroRNA-424 (322) as a new marker of disease progression in pulmonary arterial hypertension and its role in right ventricular hypertrophy by targeting SMURF1. Cardiovasc Res. 114 (1), 53–64. 10.1093/cvr/cvx187 29016730

[B7] Barroso M. Florindo C. Kalwa H. Silva Z. Turanov A. A. Carlson B. A. (2014). Inhibition of cellular methyltransferases promotes endothelial cell activation by suppressing glutathione peroxidase 1 protein expression. J. Biol. Chem. 289 (22), 15350–15362. 10.1074/jbc.M114.549782 24719327 PMC4140892

[B8] Benincasa G. Maron B. A. Affinito O. D’Alto M. Franzese M. Argiento P. (2022). Association between circulating CD4+ T cell methylation signatures of network-oriented SOCS3 gene and hemodynamics in patients suffering pulmonary arterial hypertension. J. Cardiovasc Transl. Res. 16, 17–30. 10.1007/s12265-022-10294-1 35960497 PMC9944731

[B9] Bisserier M. Mathiyalagan P. Zhang S. Elmastour F. Dorfmüller P. Humbert M. (2021). Regulation of the methylation and expression levels of the BMPR2 gene by SIN3a as a novel therapeutic mechanism in pulmonary arterial hypertension. Circulation 144 (1), 52–73. 10.1161/CIRCULATIONAHA.120.047978 34078089 PMC8293289

[B10] Donthu N. Kumar S. Mukherjee D. Pandey N. Lim W. M. (2021). How to conduct a bibliometric analysis: an overview and guidelines. J. Bus. Res. 133, 285–296. 10.1016/j.jbusres.2021.04.070

[B11] Dornas W. C. Silva M. E. (2011). Animal models for the study of arterial hypertension. J. Biosci. 36 (4), 731–737. 10.1007/s12038-011-9097-y 21857120

[B12] Durmus S. Atahan E. Kilickiran B. A. Onal B. Cakatay U. Gelisgen R. (2022). Significance of Cyclooxgenase-2 gene polymorphism and related miRNAs in pulmonary arterial hypertension. Clin. Biochem. 107, 33–39. 10.1016/j.clinbiochem.2022.06.001 35724768

[B13] Fan J. Fu A. Zhang L. (2019). Progress in molecular docking. Quant. Biol. 7, 83–89. 10.1007/s40484-019-0172-y

[B14] Friso S. Carvajal C. A. Fardella C. E. Olivieri O. (2015). Epigenetics and arterial hypertension: the challenge of emerging evidence. Transl. Res. 165 (1), 154–165. 10.1016/j.trsl.2014.06.007 25035152

[B15] Garakanidze S. Costa E. Bronze-Rocha E. Santos-Silva A. Nikolaishvili G. Nakashidze I. (2018). Methylenetetrahydrofolate reductase gene polymorphism (C677T) as a risk factor for arterial thrombosis in Georgian patients. Clin. Appl. Thromb. Hemost. 24 (7), 1061–1066. 10.1177/1076029618757345 29439641 PMC6714755

[B16] Geldsetzer P. Manne-Goehler J. Marcus M.-E. Ebert C. Zhumadilov Z. Wesseh C. S. (2019). The state of hypertension care in 44 low-income and middle-income countries: a cross-sectional study of nationally representative individual-level data from 1· 1 million adults. Lancet 394 (10199), 652–662. 10.1016/S0140-6736(19)30955-9 31327566

[B17] Ghafar M. T. A. (2020). An overview of the classical and tissue-derived renin-angiotensin-aldosterone system and its genetic polymorphisms in essential hypertension. Steroids 163, 108701. 10.1016/j.steroids.2020.108701 32717198

[B18] Guo L. Yang Y. Liu J. Wang L. Li J. Wang Y. (2014). Differentially expressed plasma microRNAs and the potential regulatory function of Let-7b in chronic thromboembolic pulmonary hypertension. PloS one 9 (6), e101055. 10.1371/journal.pone.0101055 24978044 PMC4076206

[B19] Hu F. Liu H. Wang C. Li H. Qiao L. (2022). Expression of the microRNA-30 family in pulmonary arterial hypertension and the role of microRNA-30d-5p in the regulation of pulmonary arterial smooth muscle cell toxicity and apoptosis. Exp. Ther. Med. 23 (1), 108–110. 10.3892/etm.2021.11031 34976150 PMC8674961

[B20] Improta-Caria A. C. Aras M. G. Nascimento L. De Sousa R. A. L. Aras-Júnior R. Souza B. S. F. (2021). MicroRNAs regulating renin-angiotensin-aldosterone system, sympathetic nervous system and left ventricular hypertrophy in systemic arterial hypertension. Biomolecules 11 (12), 1771. 10.3390/biom11121771 34944415 PMC8698399

[B21] Jaafar N. I. Vasudevan R. Ismail P. Abdul Aziz A. F. Mohamad N. A. Kandavello G. (2018). Analysis of angiotensin converting enzyme, endothelial nitric oxide synthase and serotonin gene polymorphisms among atrial septal defect subjects with and without pulmonary arterial hypertension. J. Cardiovasc Dev. Dis. 5 (3), 48. 10.3390/jcdd5030048 30231548 PMC6162525

[B22] Jackson K. L. Marques F. Z. Watson A. M. Palma-Rigo K. Nguyen-Huu T.-P. Morris B. J. (2013). A novel interaction between sympathetic overactivity and aberrant regulation of renin by miR-181a in BPH/2J genetically hypertensive mice. Hypertension 62 (4), 775–781. 10.1161/HYPERTENSIONAHA.113.01701 23897069

[B23] Jain S. Khera R. Girotra S. Badesch D. Wang Z. Murad M. H. (2017). Comparative effectiveness of pharmacologic interventions for pulmonary arterial hypertension: a systematic review and network meta-analysis. Chest 151 (1), 90–105. 10.1016/j.chest.2016.08.1461 27615023 PMC5310124

[B24] Jiao Y.-R. Wang W. Lei P.-C. Jia H.-P. Dong J. Gou Y.-Q. (2019). 5-HTT, BMPR2, EDN1, ENG, KCNA5 gene polymorphisms and susceptibility to pulmonary arterial hypertension: a meta-analysis. Gene 680, 34–42. 10.1016/j.gene.2018.09.020 30218748

[B25] Johnson J. Lakshmanan G. Rm V. Kalimuthu K. Sekar D. (2020). Computational identification of MiRNA-7110 from pulmonary arterial hypertension (PAH) ESTs: a new microRNA that links diabetes and PAH. Hypertens. Res. 43 (4), 360–362. 10.1038/s41440-019-0369-5 31792346

[B27] Kawai-Kowase K. Owens G. K. (2007). Multiple repressor pathways contribute to phenotypic switching of vascular smooth muscle cells. Am. J. Physiol. Cell Physiol. 292 (1), C59–C69. 10.1152/ajpcell.00394.2006 16956962

[B28] Kemp J. R. Unal H. Desnoyer R. Yue H. Bhatnagar A. Karnik S. S. (2014). Angiotensin II-regulated microRNA 483-3p directly targets multiple components of the renin–angiotensin system. J. Mol. Cell Cardiol. 75, 25–39. 10.1016/j.yjmcc.2014.06.008 24976017 PMC4157944

[B29] Khlynova O. Shishkina E. Sakhena V. Krivtsov A. Spasenkov G. Abgaryan N. (2020). TNF gene polymorphism as a risk factor that can cause arterial hypertension in patients suffering from gastroesophageal reflux disease. Health Risk Anal. (1), 126–132. 10.21668/health.risk/2020.1.14

[B30] Klimczak D. Jazdzewski K. Kuch M. (2017). Regulatory mechanisms in arterial hypertension: role of microRNA in pathophysiology and therapy. Blood Press. 26 (1), 2–8. 10.3109/08037051.2016.1167355 27177042

[B31] Kosovtseva A. Rychkova L. Kolesnikova L. Polyakov V. Bairova T. (2018). The –141c Ins/De polymorphism of the D2-dopaminergic receptor gene as a marker of early development of essential arterial hypertension in adolescents. Biomed. Biochim. Acta 3 (3), 108–115. 10.29413/abs.2018-3.3.17

[B32] Koval S. M. Snihurska I. O. Yushko K. O. Mysnychenko O. V. Penkova M. Y. Lytvynova O. M. (2020). Circulating microRNA-133a in patients with arterial hypertension, hypertensive heart disease, and left ventricular diastolic dysfunction. Front. cardiovasc Med. 7, 104. 10.3389/fcvm.2020.00104 32733920 PMC7358430

[B33] Kozomara A. Griffiths-Jones S. (2014). miRBase: annotating high confidence microRNAs using deep sequencing data. Nucleic Acids Res. 42 (D1), D68–D73. 10.1093/nar/gkt1181 24275495 PMC3965103

[B34] Lauder L. Azizi M. Kirtane A. J. Boehm M. Mahfoud F. (2020). Device-based therapies for arterial hypertension. Nat. Rev. Cardiol. 17 (10), 614–628. 10.1038/s41569-020-0364-1 32286512

[B35] Li D. Shao N.-Y. Moonen J.-R. Zhao Z. Shi M. Otsuki S. (2021a). ALDH1A3 coordinates metabolism with gene regulation in pulmonary arterial hypertension. Circulation 143 (21), 2074–2090. 10.1161/CIRCULATIONAHA.120.048845 33764154 PMC8289565

[B36] Li H. Yang Z. Gao F. Zhang Y. Meng W. Rong S. (2020). MicroRNA-17 as a potential diagnostic biomarker in pulmonary arterial hypertension. J. Int. Med. Res. 48 (6), 0300060520920430. 10.1177/0300060520920430 32600075 PMC7328490

[B37] Li P. Song J. Du H. Lu Y. Dong S. Zhou S. (2021b). MicroRNA-663 prevents monocrotaline-induced pulmonary arterial hypertension by targeting TGF-β1/smad2/3 signaling. J. Mol. Cell Cardiol. 161, 9–22. 10.1016/j.yjmcc.2021.07.010 34339758

[B38] Li Q. Wu J. Xu Y. Liu L. Xie J. (2019). Role of RASEF hypermethylation in cigarette smoke-induced pulmonary arterial smooth muscle remodeling. Respir. Res. 20, 52–14. 10.1186/s12931-019-1014-1 30845941 PMC6407244

[B39] Lv Y. Fu L. Zhang Z. Gu W. Luo X. Zhong Y. (2019). Increased expression of MicroRNA‐206 inhibits potassium voltage‐gated channel subfamily a member 5 in pulmonary arterial smooth muscle cells and is related to exaggerated pulmonary artery hypertension following intrauterine growth retardation in rats. J. Am. Heart Assoc. 8 (2), e010456. 10.1161/JAHA.118.010456 30636484 PMC6497345

[B40] Ma W. Qiu Z. Bai Z. Dai Y. Li C. Chen X. (2021a). Inhibition of microRNA-30a alleviates vascular remodeling in pulmonary arterial hypertension. Mol. Ther. Nucleic Acids 26, 678–693. 10.1016/j.omtn.2021.09.007 34703652 PMC8517099

[B41] Ma Y. Chen S. S. Jiang F. Ma R. Y. Wang H. L. (2021b). Bioinformatic analysis and validation of microRNA‐508‐3p as a protective predictor by targeting NR4A3/MEK axis in pulmonary arterial hypertension. J. Cell Mol. Med. 25 (11), 5202–5219. 10.1111/jcmm.16523 33942991 PMC8178270

[B42] Mao M. Song S. Li X. Lu J. Li J. Zhao W. (2023). Advances in epigenetic modifications of autophagic process in pulmonary hypertension. Front. Immunol. 14, 1206406. 10.3389/fimmu.2023.1206406 37398657 PMC10313199

[B43] Marushchak M. Maksiv K. Krynytska I. (2019a). ACE gene I/D polymorphism and arterial hypertension in patients with COPD. Pneumologia 68 (3), 114–119. 10.2478/pneum-2019-0039

[B44] Marushchak M. Maksiv K. Krynytska I. Kozak K. (2019b). Association between arterial hypertension and chronic obstructive pulmonary disease: role of AGT gene polymorphism. Pneumologia 68 (4), 174–182. 10.2478/pneum-2019-0036

[B45] Massignam E. T. Dieter C. Assmann T. S. Duarte G. C. K. Bauer A. C. Canani L. H. (2022). The rs705708 A allele of the ERBB3 gene is associated with lower prevalence of diabetic retinopathy and arterial hypertension and with improved renal function in type 1 diabetic patients. Microvasc. Res. 143, 104378. 10.1016/j.mvr.2022.104378 35594935

[B46] Mills K. T. Bundy J. D. Kelly T. N. Reed J. E. Kearney P. M. Reynolds K. (2016). Global disparities of hypertension prevalence and control: a systematic analysis of population-based studies from 90 countries. Circulation 134 (6), 441–450. 10.1161/CIRCULATIONAHA.115.018912 27502908 PMC4979614

[B48] Mohamed R. H. Elzaiat A. M. Elfeky H. K. Al-Karamany A. S. (2022). Polymorphism of 11 β-hydroxysteroid dehydrogenase Type 1 gene in hypertensive patients. Zagazig Univ. Med. J. 28 (4), 728–733. 10.21608/ZUMJ.2021.42702.1942

[B49] Moskalenko M. Ponomarenko I. Milanova S. Verzilina I. Efremova O. Polonikov A. (2020). Polymorphic locus rs1061624 of the ТNFR2 gene is associated with the development of arterial hypertension in males. Kardiologiia 60 (8), 78–83. 10.18087/cardio.2020.8.n996 33155962

[B50] Napoli C. Benincasa G. Loscalzo J. (2019). Epigenetic inheritance underlying pulmonary arterial hypertension: a new challenge for network medicine. Arterioscler. Thromb. Vasc. Biol. 39 (4), 653–664. 10.1161/ATVBAHA.118.312262 30727752 PMC6436974

[B51] Napoli C. Grimaldi V. De Pascale M. R. Sommese L. Infante T. Soricelli A. (2016). Novel epigenetic-based therapies useful in cardiovascular medicine. World J. Cardiol. 8 (2), 211–219. 10.4330/wjc.v8.i2.211 26981216 PMC4766271

[B52] Nie X. Chen Y. Tan J. Dai Y. Mao W. Qin G. (2019). MicroRNA-221-3p promotes pulmonary artery smooth muscle cells proliferation by targeting AXIN2 during pulmonary arterial hypertension. Vasc. Pharmacol. 116, 24–35. 10.1016/j.vph.2017.07.002 28694128

[B53] Nikulina S. Y. Chernova A. A. Tolstokorova Y. A. Varavko Y. O. (2022). Associative role of polymorphism of the gene of <i&gt;MMP-9&lt;/i&gt; (rs11697325) in development of arterial hypertension in patients with the rheumatoid arthritis. CardioSomatics 13 (2), 76–80. 10.17816/cs110917

[B54] Nunes I. F. Cavalcante A. A. Alencar M. V. Carvalho M. D. Sarmento J. L. Teixeira N. S. (2020). Meta-analysis of the association between the rs228570 vitamin D receptor gene polymorphism and arterial hypertension risk. Adv. Nutr. 11 (5), 1211–1220. 10.1093/advances/nmaa076 32597926 PMC7490169

[B56] Ou H. Liu D. Zhao G. Gong C. Li Y. Zhao Q. (2022). Association between AT1 receptor gene polymorphism and left ventricular hypertrophy and arterial stiffness in essential hypertension patients: a prospective cohort study. BMC Cardiovasc Disord. 22 (1), 571. 10.1186/s12872-022-03024-7 36577936 PMC9795750

[B57] Ou M. Zhang C. Chen J. Zhao S. Cui S. Tu J. (2019). Overexpression of microRNA-340-5p inhibits pulmonary arterial hypertension induced by acute pulmonary embolism by down-regulating the expression of inflammatory factors interleukin-1β and interleukin-6. EBioMedicine 21, 542–554. 10.1016/j.omtn.2020.05.022

[B58] Owens G. K. Kumar M. S. Wamhoff B. R. (2004). Molecular regulation of vascular smooth muscle cell differentiation in development and disease. Physiol. Rev. 84 (3), 767–801. 10.1152/physrev.00041.2003 15269336

[B59] Pavlova O. Ogurtsova S. Liventseva M. Korobko I. Y. Mrochek A. (2018). Arterial hypertension and methylenetetrahydrofolate reductase C677T gene polymorphism. Kardiologiia 17 (10), 5–11. 10.18087/cardio.2018.10.10161 30359211

[B60] Pinto I. Martins D. (2017). Prevalence and risk factors of arterial hypertension: a literature review. J. Cardiovasc Med. Ther. 2, 5300–6121.

[B61] Pokoeva Z. Pushkarev B. Bolshakova O. Ilyamakova N. Vitkovsky Y. A. (2022). Research of the rs11064153 variant of the SCNN1A gene in patients with arterial hypertension and in healthy people in the Trans-Baikal. Arter. Hypertens. 28 (5), 593–599. 10.18705/1607-419x-2022-28-5-593-599

[B62] Prohaska C. C. Zhang X. Schwantes-An T.-H. Stearman R. Aldred M. A. Lutz K. (2022). Rasa3 is a novel candidate gene in sickle cell disease-associated pulmonary hypertension and pulmonary arterial hypertension. Circulation 146 (1), A13301–A. 10.1161/circ.146.suppl_1.13301 PMC10124178 37101805

[B63] Raina R. Krishnappa V. Das A. Amin H. Radhakrishnan Y. Nair N. R. (2019). Overview of monogenic or mendelian forms of hypertension. Front. Pediatr. 7, 263. 10.3389/fped.2019.00263 31312622 PMC6613461

[B64] Rao H. Wu H. Yu Z. Huang Q. (2022). APOE genetic polymorphism rs7412 T/T genotype may be a risk factor for essential hypertension among Hakka people in southern China. Int. J. Hypertens. 2022, 8145896. 10.1155/2022/8145896 36158751 PMC9492438

[B65] Rehmsmeier M. Steffen P. Höchsmann M. Giegerich R. (2004). Fast and effective prediction of microRNA/target duplexes. Rna 10 (10), 1507–1517. 10.1261/rna.5248604 15383676 PMC1370637

[B67] Russomanno G. Jo K. B. Abdul-Salam V. B. Morgan C. Alzaydi M. Wilkins M. R. (2020). miR-150-PTPMT1-cardiolipin signaling in pulmonary arterial hypertension. Mol. Ther. Nucleic Acids 23, 142–153. 10.1016/j.omtn.2020.10.042 33335799 PMC7733016

[B68] Sayers E. W. Beck J. Bolton E. E. Bourexis D. Brister J. R. Canese K. (2021). Database resources of the national center for biotechnology information. Nucleic Acids Res. 49 (D1), D10–D17. 10.1093/nar/gkaa892 33095870 PMC7778943

[B70] Shannon P. Markiel A. Ozier O. Baliga N. S. Wang J. T. Ramage D. (2003). Cytoscape: a software environment for integrated models of biomolecular interaction networks. Genome Res. 13 (11), 2498–2504. 10.1101/gr.1239303 14597658 PMC403769

[B71] Shi C. Liu X. Rahman J. U. Liu G. Jiang Y. (2022). MicroRNA-144-3p controls the apoptosis of pulmonary artery endothelial cells in pulmonary arterial hypertension via the BMPR2/Smad4 signaling pathway. Clin. Transl. Disc 2 (1), e27. 10.1002/ctd2.27

[B72] Shishkina E. Khlynova O. Vasilets L. Sakhena V. Krivtsov A. (2019). Role of C807T polymorphism of ITGA2 gene of collagen receptor and platelet aggregation activity in patients with arterial hypertension. Kazan Med. J. 100 (3), 386–391. 10.17816/kmj2019-386

[B73] Shnayder N. A. Petrova M. M. Moskaleva P. V. Shesternya P. A. Pozhilenkova E. A. Nasyrova R. F. (2021). The role of single-nucleotide variants of NOS1, NOS2, and NOS3 genes in the comorbidity of arterial hypertension and tension-type headache. Molecules 26 (6), 1556. 10.3390/molecules26061556 33809023 PMC8002043

[B74] Sofronova S. Romanova A. Kirillina M. (2019b). Correlation of the M235T polymorphism of the AGT gene with arterial hypertension and its risk factors in the indigenous people of the Arctic Territory of Yakutia. Yakut Med. J. (3), 16–19. 10.25789/ymj.2019.67.04

[B75] Sofronova S. I. Kirillina M. P. Kononova I. V. Romanova A. N. Nikolaev V. M. Kononova S. K. (2019a). Association of the AGT gene m235t (rs699) polymorphism with arterial hypertension and metabolic risk factors in the indigenous people of Yakutia. Int. J. Biomed. 9 (4), 287–291. 10.21103/article9(4)_oa2

[B77] Suzuki H. Kataoka M. Hiraide T. Aimi Y. Yamada Y. Katsumata Y. (2018). Genomic comparison with supercentenarians identifies RNF213 as a risk gene for pulmonary arterial hypertension. Circ. Genom Precis. Med. 11 (12), e002317. 10.1161/CIRCGEN.118.002317 30562119

[B78] Synetos A. Toutouzas K. Stathogiannis K. Latsios G. Tsiamis E. Tousoulis D. (2013). MicroRNAs in arterial hypertension. Curr. Top. Med. Chem. 13 (13), 1527–1532. 10.2174/15680266113139990101 23745804

[B79] Takagi K. Kawamoto M. Higuchi T. Tochimoto A. Harigai M. Kawaguchi Y. (2020). Single nucleotide polymorphisms of the HIF1A gene are associated with susceptibility to pulmonary arterial hypertension in systemic sclerosis and contribute to SSc‐PAH disease severity. Int. J. Rheum. Dis. 23 (5), 674–680. 10.1111/1756-185X.13822 32144871

[B80] Takeda Y. Demura M. Yoneda T. Takeda Y. (2021). DNA methylation of the angiotensinogen gene, AGT, and the aldosterone synthase gene, CYP11B2 in cardiovascular diseases. Int. J. Mol. Sci. 22 (9), 4587. 10.3390/ijms22094587 33925539 PMC8123855

[B81] Topchieva L. Balan O. Korneeva V. Malysheva I. (2019). The role of inducible NOS2 gene polymorphism in the development of essential arterial hypertension. Exp. Biol. Med. 168, 79–83. 10.1007/s10517-019-04652-4 31768780

[B82] Van Eck N. Waltman L. (2010). Software survey: VOSviewer, a computer program for bibliometric mapping. scientometrics 84 (2), 523–538. 10.1007/s11192-009-0146-3 20585380 PMC2883932

[B83] Velloso L. A. Folli F. Sun X. J. White M. F. Saad M. Kahn C. R. (1996). Cross-talk between the insulin and angiotensin signaling systems. Proc. Natl. Acad. Sci. 93 (22), 12490–12495. 10.1073/pnas.93.22.12490 8901609 PMC38019

[B84] Villar A. B. Valverde D. Lago M. (2019). Functional study of polymorphisms in the promoter region of the endothelin-1 gene in pulmonary arterial hypertension. Eur. Respir. J. 54, PA5043. 10.1183/13993003.congress-2019.PA5043

[B85] Vivsiana I. Habor H. Marushchak M. (2021). The role of the IRS1 gene (rs2943640) in the comorbid course of type 2 diabetes mellitus, obesity and arterial hypertension. J. Phys. Educ. Sport 11 (1), 388–399. 10.12775/jehs.2021.11.1.039

[B86] Wang C. Xu G. Wen Q. Peng X. Chen H. Zhang J. (2019). CBS promoter hypermethylation increases the risk of hypertension and stroke. Clinics 74, e630. 10.6061/clinics/2019/e630 30916171 PMC6438132

[B87] Wang H.-J. Lo W.-Y. Lin L.-J. (2013). Angiotensin-(1–7) decreases glycated albumin-induced endothelial interleukin-6 expression via modulation of miR-146a. Biochem. Biophys. Res. Commun. 430 (3), 1157–1163. 10.1016/j.bbrc.2012.12.018 23246834

[B88] Wang Q. Sun Y. Zhao Q. Wu W. Wang L. Miao Y. (2022). Circular RNAs in pulmonary hypertension: emerging biological concepts and potential mechanism. Animal Models Exp. Med. 5 (1), 38–47. 10.1002/ame2.12208 PMC8879624 35229989

[B89] Wang Y. Xu X. Huo Y. Liu D. Cui Y. Liu Z. (2015). Predicting hyperhomocysteinemia by methylenetetrahydrofolate reductase C677T polymorphism in Chinese patients with hypertension. Clin. Appl. Thromb. Hemost. 21 (7), 661–666. 10.1177/1076029613519849 24459043

[B90] Whelton P. K. Flack J. M. Jennings G. Schutte A. Wang J. Touyz R. M. (2023). Editors’ commentary on the 2023 ESH management of arterial hypertension guidelines. Hypertension 80 (9), 1795–1799. 10.1161/HYPERTENSIONAHA.123.21592 37354199 PMC10527435

[B92] Wulandari A. S. Widodo M. A. Sardjono T. W. Lyrawati D. (2018). Angiotensinogen II Type I receptor A1166C is associated with serum sodium level and essential hypertension in Javanese population. Indian J. Public Health 9 (12), 415. 10.5958/0976-5506.2018.01872.7

[B93] Yang S. Gao Y. Liu G. Li J. Shi K. Du B. (2015). The human ATF1 rs11169571 polymorphism increases essential hypertension risk through modifying miRNA binding. FEBS Lett. 589 (16), 2087–2093. 10.1016/j.febslet.2015.06.029 26149214

[B94] Yapijakis C. Papakosta V. Vassiliou S. (2019). ACE gene variant causing high blood pressure may be associated with medication-related jaw osteonecrosis. vivo 33 (2), 559–562. 10.21873/invivo.11510 PMC6506304 30804141

[B95] Yang C. Rong R. Li Y. Cheng M. Luo Y. (2022). Decrease in LINC00963 attenuates the progression of pulmonary arterial hypertension via microRNA-328-3p/profilin 1 axis. J. Clin. Lab. Anal. 36 (5), e24383. 10.1002/jcla.24383 35349725 PMC9102517

[B96] Yang H. Lu Y. Yang H. Zhu Y. Tang Y. Li L. (2021). Integrated weighted gene co-expression network analysis uncovers STAT1 (signal transducer and activator of transcription 1) and IFI44L (interferon-induced protein 44-like) as key genes in pulmonary arterial hypertension. Bioengineered 12 (1), 6021–6034. 10.1080/21655979.2021.1972200 34516357 PMC8806536

[B97] Yermolenko S. Chumachenko Y. Orlovskyi V. Moiseyenko I. Orlovskyi O. (2021). The association between Gly460Trp-polymorphism of Alpha-Adducin 1 Gene (ADD1) and arterial hypertension development in Ukrainian population. Int. J. Hypertens. 2021, 5596974–5596979. 10.1155/2021/5596974 34055401 PMC8112959

[B98] Yu H. Xu M. Dong Y. Liu J. Li Y. Mao W. (2018). 1, 25 (OH) 2D3 attenuates pulmonary arterial hypertension via microRNA-204 mediated Tgfbr2/Smad signaling. Exp. Cell Res. 362 (2), 311–323. 10.1016/j.yexcr.2017.11.032 29196166

[B99] Yushko K. Koval S. Snihurska I. Mysnychenko O. (2020). The associations of circulating microRNA-133a with parameters of hypertension heart disease in patients with arterial hypertension and obesity. Eur. Heart J. 41 (2), ehaa946. 10.1093/ehjci/ehaa946.2712

[B100] Zhang W. Li Y. Xi X. Zhu G. Wang S. Liu Y. (2020). MicroRNA-15a-5p induces pulmonary artery smooth muscle cell apoptosis in a pulmonary arterial hypertension model via the VEGF/p38/MMP-2 signaling pathway. Int. J. Mol. Med. 45 (2), 461–474. 10.3892/ijmm.2019.4434 31894295 PMC6984778

[B101] Zhou Y. Jiang Y. Chen S. J. (2022). RNA–ligand molecular docking: advances and challenges. Wiley Interdiscip. Rev. Comput. Mol. Sci. 12 (3), e1571. 10.1002/wcms.1571 37293430 PMC10250017

[B102] Zhu N. Li P. Du H. Qin Y. Zhao X. (2020). MicroRNA-663 ameliorates monocrotaline-induced pulmonary arterial hypertension by targeting tgfβ1/smad2/3 signaling. Circulation 142 (3), A12535–A. 10.1161/circ.142.suppl_3.12535

[B103] Zhu T. T. Zhang W. F. Yin Y. L. Liu Y. H. Song P. Xu J. (2019). MicroRNA-140-5p targeting tumor necrosis factor-α prevents pulmonary arterial hypertension. J. Cell Physiol. 234 (6), 9535–9550. 10.1002/jcp.27642 30367500

